# Effects of Continuous Exposure to Yellow Light on the Behavior and Longevity of *Anomala corpulenta*

**DOI:** 10.3390/insects17040394

**Published:** 2026-04-04

**Authors:** Yueli Jiang, Xiaoguang Liu, Zhongjun Gong, Yuqing Wu, Li Qiao, Ruijie Lu, Jing Zhang, Jin Miao, Tong Li

**Affiliations:** 1 Institute of Plant Protection, Henan Key Laboratory of Agricultural Pest Monitoring and Control, Key Laboratory of Integrated Crop Pests Management on Crops in Southern Region of North China, Ministry of Agriculture and Rural Affairs, Henan Academy of Agricultural Sciences, Zhengzhou 450002, China; yueli006@126.com (Y.J.);; 2 Henan International Laboratory for Green Pest Control, Henan Engineering Laboratory of Pest Biological Control, College of Plant Protection, Henan Agricultural University, Zhengzhou 450046, China; xgliu2000@aliyun.com; 3 College of Agriculture, Xinyang Agriculture and Forestry University, Xinyang 464000, China

**Keywords:** *Anomala corpulenta*, emergence rhythm, feeding behavior, mating behavior, light control

## Abstract

*Anomala corpulenta* (Motschulsky, 1854) (Coleoptera: Scarabaeidae), an important agricultural and forestry pest, is a beetle widely distributed in many countries, inflicting damage on numerous crops. Given the limited selectivity of commonly used light trapping devices for insects and their potential adverse effects on the ecological environment, there is a pressing need for innovative light control methods. This study investigates the effects of continuous exposure to yellow light on the behavioral activities of *A. corpulenta* adults, which are nocturnal. The experimental setup comprised a light experimental group (exposed continuously to yellow light at wavelengths of 565–585 nm and intensities of 30–40 lx at night) and a control group (kept in a dark room). Observations were made on emergence, mating, feeding, and mortality. Results showed that continuous exposure to yellow light significantly alters the emergence rhythm of *A. corpulenta*, leading to delays and dispersions in peak emergence, with emergence occurring during the light period. The emergence rates varied significantly from the control group during specific periods, and the overall emergence rate was notably affected, with female insects exhibiting greater sensitivity. Furthermore, food consumption and the number of mating pairs were significantly lower compared to the control group. Continuous exposure to yellow light also influenced the longevity of *A. corpulenta*; in the mixed test group, female insects had a lifespan of 20 days, while males lived for 18 days. In the sexually isolated test group, both sexes died within 16 days, with the survival rates of the experimental group being lower than those of the control group on certain days. This study concludes that continuous exposure to yellow light significantly modifies the emergence rhythm of *A. corpulenta*, while reducing the emergence rate, total food intake, and the number of mating pairs. Notably, in the mixed-sex test group, the survival probability of females in the experimental group was significantly lower than that of the control group. These findings provide a theoretical foundation for the light control of *A. corpulenta* and contribute to the field of insect visual ecology.

## Simple Summary

Continuous nighttime exposure to yellow light (565–585 nm, 30–40 lx) significantly disrupted key behavioral activities in the nocturnal beetle *Anomala corpulenta* (Motschulsky, 1854) (Coleoptera: Scarabaeidae), a major agricultural pest. Treated adults exhibited delayed and dispersed emergence peaks, with emergence occurring during the light period, alongside significant reductions in feeding, mating, and longevity. Notably, females demonstrated greater sensitivity to light treatment than males. These findings underscore the potential of targeted spectral lighting as an environmentally sustainable strategy for pest management and contribute to our understanding of insect visual ecology.

## 1. Introduction

*Anomala corpulenta* (Motschulsky, 1854) is an important agricultural and forestry pest beetle in the Rutelinae family (Coleoptera: Scarabaeidae). It is widely distributed throughout China, Japan, North Korea, Southeast Asia, and other countries. Its larvae damage the roots or pods of many crops including corn, peanuts, soybeans, and wheat, resulting in direct or indirect economic losses. Adults often gather and gorge on leaves, damaging many forest and fruit trees such as elm, poplar, and grape, seriously affecting the yield and quality of crops [1,2,3]. Currently, chemical control remains the primary approach to pest management; however, it has resulted in significant issues such as pest resistance, ecosystem pollution, and risks to the quality and safety of agricultural products. In the context of strategically advancing green agricultural development, the establishment of a novel ecological regulation-based pest control technology system has become an urgent necessity for ensuring both food security and ecological integrity.

Light-induced pest control technology, due to its environmental friendliness, high efficiency, and convenience, has emerged as a leading area of research in global pest control. This technology is based on the phototaxis mechanism of insects, which regulates their behavior in response to light. Its aim is to attract or repel adult pests using specific light wavelengths, thereby achieving precise control over pest populations. The adults of *A. corpulenta* exhibit strong phototaxis and, in addition to conventional chemical control, light trapping is an important measure for adult control. Commonly used light trapping devices use ultraviolet light such as black light and include frequency-vibrating insecticidal lamps, or mixed light sources such as high-pressure mercury lamps, incandescent lamps, and searchlights. However, these traps are non-specific and, besides killing pests, they also kill natural enemies and non-target insects while affecting plants and the ecological environment to varying degrees [4,5,6]. A study conducted by Delaware State University revealed that among the 13,789 insect species captured by black light traps, only 31 were target mosquito species, representing a mere 0.22%. In contrast, 13.5% of the captured species were natural enemy insects, and 48.4% were aquatic non-pest species, resulting in a beneficial-to-harmful ratio as high as 63:1. This finding significantly disrupts the balance of biodiversity [4]. Additionally, ultraviolet and mixed light lamps exert substantial effects on plants, as UV spectra interfere with plant photomorphogenesis and the regulation of photoperiods [6], thereby creating secondary ecological risks. This model of ‘broad-spectrum trapping fundamentally contradicts the principles of precision plant protection; therefore, new and novel environmentally friendly light control traps are needed for the control of *A. corpulenta*.

Insects perceive light waves mainly through the visual pigments in their compound eyes, where most insects have two types of visual pigments. One pigment can accept green-yellow light with wavelengths of about 550 nm, and the other pigment accepts ultraviolet-blue-violet light with wavelengths below 480 nm [7]. Differences in visual pigments provide the physiological basis for the diversity of light-responsive behaviors observed among various insect species. Based on the spectral response characteristics, current pest light control systems have evolved two primary technical approaches. Black light or frequency-vibrating insecticidal lamps specifically target the ultraviolet-blue light receptors in the compound eyes of insects, whereas yellow light (moth-proof lamps) leverages the biological function of the green-yellow light receptors. The compound eyes of nocturnal moths are in a “light adaptation” state, also known as “bright eyes” during the day, and in a “dark adaptation” state, also known as “dark eyes” at night. Feeding, mating, and oviposition of nocturnal moths happen in the “dark adaptation” state at night. When sufficient yellow light is present at night, some moths remain in the “light adaptation” state—negatively affecting feeding and reproduction and thus controlling pest density. For example, the screening pigments of the compound eyes of *Spodoptera exigua* cover the compound eyes during yellow light exposure, similar to that during the day (light adaptation state) [8,9]. This approach disrupts key behavioral rhythms such as feeding, mating, and oviposition. Beyond the effects on moths, light of varying wavelengths has been shown to influence the behavior of a wide range of insect orders. For dipteran pests, studies have demonstrated that specific wavelengths can modulate phototactic responses. Research on the oriental fruit fly (*Bactrocera dorsalis*) revealed that both males and females exhibit significant preference for yellow (572 nm) and green light (536 nm), while showing weaker responses to red, purple, and blue light [10]. Furthermore, exposure to green light at night was found to significantly increase the emergence rate of *B. dorsalis*; However, it notably, shortened adult longevity [11]. Among coleopteran pests, the red flour beetle (*Tribolium castaneum*) provides an interesting case of visual system specialization. Genomic studies have revealed that this species encodes only UV-opsin and long-wavelength (LW)-opsin, lacking the blue-sensitive photoreceptor (B-opsin) found in many other insects [12]. Behavioral studies subsequently confirmed sex- and strain-specific spectral preferences in *T. castaneum*, with females showing greater attraction to blue-green and green light compared to UV, blue, and red light, while males exhibited no significant differences across wavelengths [13]. These findings underscore the species-specific nature of spectral sensitivity and the importance of targeted investigations for each key pest species.

When developing spectral control strategies, it is essential to consider ecological interaction effects holistically. Plant photosynthetic systems primarily respond to blue (400–500 nm) and red light (600–700 nm) wavelengths [6], while insect visual sensitivity is concentrated in the ultraviolet and yellow-green spectral bands [7]. This mismatch in spectral requirements provides yellow-light control technology distinct advantages: it mitigates the risks of photosynthetic inhibition while effectively regulating pest populations through targeted interference with their visual–behavioral coupling system. This technical system has established a mature standardized application model in Japan [9]. Through systematic research, our team has innovatively developed a yellow-light pest control parameter system tailored to China’s agricultural ecology [14,15,16,17,18,19,20,21], which has shown promising results in field validations across major agricultural production regions [22,23]. In addition to direct behavioral manipulation, light exposure can profoundly impact insect physiology and population dynamics through sublethal effects. For instance, continuous light exposure has been reported to disrupt the circadian rhythms of insects, leading to desynchronized emergence patterns and altered reproductive strategies. Research on the pea aphid (*Acyrthosiphon pisum*) has elucidated the molecular components and neural architecture of its circadian rhythm, which is intricately linked to the neurohormonal system regulating aphid reproduction [24]. Furthermore, exogenous melatonin treatment under long-day conditions has been shown to induce the production of males and intermediate females in pea aphids—characteristics typically associated with short-day conditions demonstrating that light signals can influence reproductive mode through biochemical pathways [25]. Additionally, environmental stresses, including thermal stress, have been shown to induce oxidative stress responses in predatory ladybird beetles (*Harmonia axyridis*), altering antioxidant enzyme activities and potentially affecting longevity and reproductive output [26]. These findings suggest that light exposure, as an environmental stressor, may similarly impact insect physiology through multiple pathways, making it a potentially powerful tool for disrupting essential life history traits beyond simple attraction or repulsion.

It is assumed that, since both the adults of *A. corpulenta* and moths are nocturnal, yellow light could also interfere with the daily rhythms of these pests, thereby controlling the pest population [14,15,16,17,18,19,20,21]. However, there are significant differences between adult beetles and moths in terms of their visual systems, behavioral ecology, and activity patterns. The visual response patterns of these insects to yellow light, along with their adaptive changes following prolonged exposure, remain unclear. Previous studies found that both yellow and green light irradiation could affect the emergence rhythm, feeding, and mating behaviors of *A. corpulenta* [21]. The results preliminarily confirm that the light of specific wavelengths has the potential to regulate the behavior of *A. corpulenta*. Unfortunately, the study only lasted for one day, and evidence supporting the use of yellow light to control *A. corpulenta* was insufficient. Prolonged exposure to light may induce physiological adaptations or behavioral compensations in insects, which could reduce the efficacy of control measures. Therefore, the primary objective of this study is to systematically investigate the effects of continuous yellow light exposure on the behavioral activities and the longevity of *A. corpulenta* adults. Specifically, we aim to: (1) determine the impact on the adult emergence rhythm and rate; (2) quantify the changes in feeding and mating behaviors; and (3) assess the effects on adult longevity and survival probability under both sexually isolated and mixed-sex conditions. By addressing these objectives, this study seeks to provide a theoretical basis for the application of yellow light in the integrated management of *A. corpulenta* and to contribute to a deeper understanding of insect visual ecology.

## 2. Materials and Methods

### 2.1. Test Insects

Adult *A. corpulenta* were collected from the Yuanyang Experimental Base of the Henan Academy of Agricultural Sciences (113.46° E, 35.08° N). The collected adults were temporarily reared in indoor glass tanks and provided with fresh poplar leaves as food, with the temperature controlled at 25~28 °C and relative humidity maintained at approximately 70~75%. The experiments were conducted from June to July 2024.

### 2.2. Experimental Equipment and Lights

A 10 W yellow LED lamp (wavelength: 565~585 nm) was customized for this study by Hebi Guoli Optoelectronic Technology Co., Ltd. (Hebi, China). An illuminometer (model: TES-1334A) was purchased from TES Electrical Electronic Corp., Taiwan, China, and acrylic boxes (30 cm × 20 cm × 20 cm) were obtained from Daize Acrylic Factory Store.

### 2.3. Experimental Methods

Healthy male and female individuals were selected as test subjects and placed in transparent acrylic boxes. A layer of soil about 5 cm thick was added to the bottom of each box, and several poplar leaves cut into rectangles with an area of about 200 cm^2^ were placed on the soil as a food source. In the light experimental groups, a yellow light tube was suspended above the acrylic box, providing a measured light intensity of 30~40 lx. In the control group, the box was placed in a dark room, maintaining a measured light intensity of 0 lx. Control populations were kept until all individuals succumbed. In the female-male isolated test groups, 20 females or 20 males were used per treatment, whereas in the female-male mixed test groups, 10 females and 10 males were employed per treatment, with each treatment repeated a minimum of three times.

From 20:00 to 6:00 the following morning, yellow light irradiation was administered to the experimental groups, while normal sunlight irradiation with a light intensity of approximately 1000 lx, was applied from 6:00 to 20:00. For the control group, complete darkness was maintained from 20:00 to 6:00 the next morning, with normal sunlight irradiationallowed from 6:00 to 20:00. Behavioral observations were made every half hour, both day and night, utilizing weak red light for illumination during nocturnal observations. The number of emerging insects and mating pairs was documented, and the area of leaves consumed was determined at the end of the experiment. The number of deceased test insects was counted daily and subsequently removed. The entire experiment was conducted in an artificial climate chamber to ensure stable environmental conditions. During the experiment, the temperature was maintained at 25 to 28 °C, with the relative humidity set at 70 to 75%.

### 2.4. Data Analysis

Data were sorted and analyzed using Excel. Statistical analyses were performed using DPS 9.05 and GraphPad Prism 10.1.2. All percentage data were transformed using the arcsine-square-root method prior to analysis. For comparisons involving four replicates (*n* = 4), independent samples *t*-tests were validated through exact permutation tests. In cases with three replicates (*n* = 3), exact permutation tests yielding *p* = 0.10 were interpreted as indicative of complete separation representing the minimum achievable value for *n*_1_ = *n*_2_ = 3. Cohen’s d effect sizes were calculated for all comparisons, with *d* > 0.8 classified as a large effect size. Significance was determined based on a combination of these methods, with primary emphasis placed on exact permutation tests for *n* = 4 and on complete separation combined with effect sizes for *n* = 3. Survival curves were generated using the Kaplan–Meier method, and group differences were assessed using the Log-rank (Mantel–Cox) test. Graphs were created using GraphPad Prism version 10.1.2.

## 3. Results

### 3.1. Effects of Continuous Yellow Light Exposure on the Emergence Rate and Rhythm of A. corpulenta

**Figure 1:**
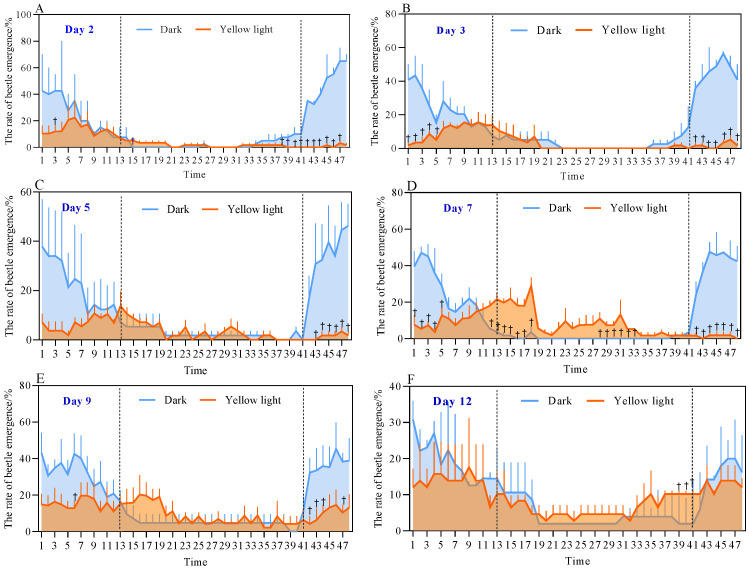
Emergence rate and rhythm of female *Anomala corpulenta* (female-male isolation test group). Panels (**A**–**F**) show the emergence rates for the yellow light-treated group (orange) and the control group (blue). Data are shown as mean ± SE. The shaded area under the curve is for visual guidance. Dashed vertical lines separate different phases of emergence rhythm.Values 1, 2 … 47, 48 on the horizontal axis represent time points from 00:00, 00:30 … to 23:00, 23:30, respectively. Daggers (†) indicate complete separation between treatment and control groups (exact permutation test, *p* = 0.10, the minimum achievable value for *n* = 3). For all time points marked with a dagger, effect sizes were large (Cohen’s *d* > 0.8), indicating biologically meaningful effects.

**Figure 2:**
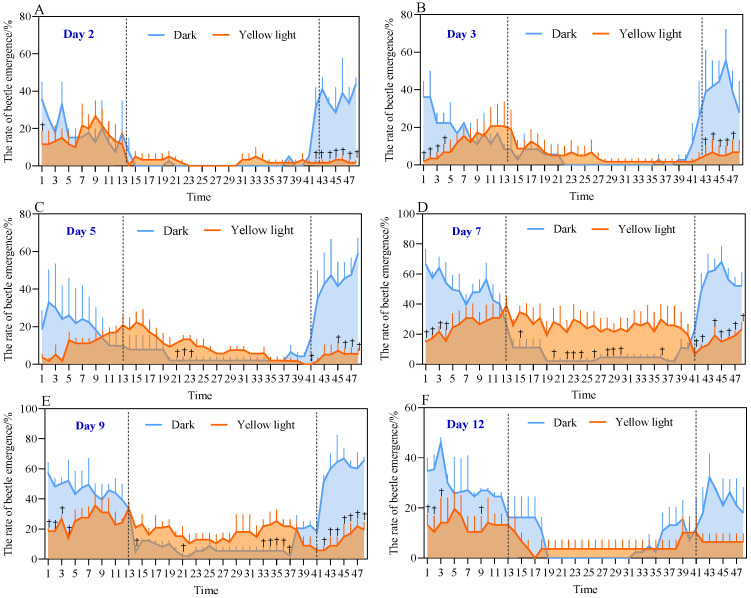
Emergence rate and rhythm of male *Anomala corpulenta* (female-male isolation test group). Panels (**A**–**F**) show the emergence rates for the yellow light-treated group (orange) and the control group (blue). Data are shown as mean ± SE (*n* = 3). The shaded area under the curve is for visual guidance. Dashed vertical lines separate different phases of emergence rhythm. Values 1, 2 … 47, 48 on the horizontal axis represent time points from 00:00, 00:30 … to 23:00, 23:30, respectively. Daggers (†) indicate complete separation between treatment and control groups (exact permutation test, *p* = 0.10, the minimum achievable value for *n* = 3). For all time points marked with a dagger, effect sizes were large (Cohen’s *d* > 0.8), indicating biologically meaningful effects.

Figure 1 and Figure 2 illustrate the emergence rate and rhythm of the male and female isolation test groups in *A. corpulenta*. There was an obvious emergence peak during the 2nd–5th days of the experiment between 00:00–06:00, which was significantly earlier than that of the control group between 20:00–24:00 (Figure 1 and Figure 2A–C). The overall emergence rate of the experimental group was also lower than that of the control group (Figure 2A–E). After the 6th day, the emergence rate of the experimental group did not change significantly and they even emerged during the normal daylight period (Figure 1 and Figure 2D–F). For each of the 48 time points across all subplots in Figure 1 and Figure 2 (*n* = 3), the differences between treatment and control groups were evaluated using three complementary statistical approaches. Independent samples *t*-tests revealed significant differences (*p* < 0.05) at a limited number of time points, primarily during the early phase of the experiment. Exact permutation tests identified a slightly larger set of time points where the treatment and control groups exhibited complete separation (exact *p* = 0.10, the minimum achievable value for *n* = 3), indicating a clear distinction between the two groups despite the small sample size. Furthermore, Cohen’s d effect sizes showed that approximately half of all time points had large effect sizes (Cohen’s *d* > 0.8), demonstrating that the treatment had a biologically meaningful impact on emergence behavior across a substantial portion of the observation period.

Collectively, these results indicate that the experimental group of *A. corpulenta* significantly adjusted its emergence rhythm and rate with an increasing number of irradiation days: the emergence peak was delayed, more dispersed, with multiple peaks and even emergence during the light period (Figure 1 and Figure 2).

**Figure 3:**
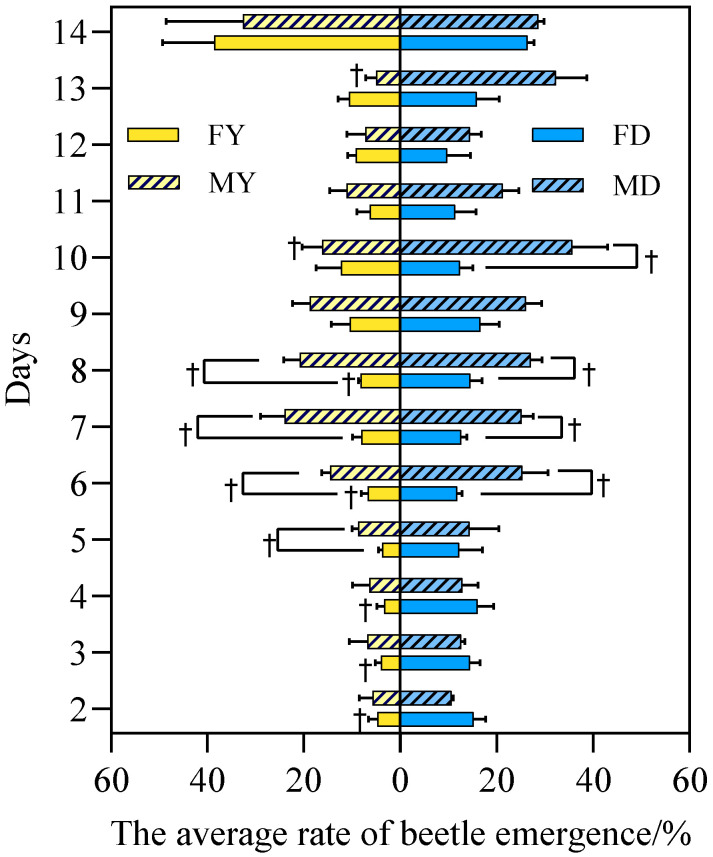
Mean emergence rate of male and female *Anomala corpulenta* (female-male isolation test group). Y: yellow light; D: dark; F: female; M: male. Data are shown as mean ± SE. Daggers (†) indicate complete separation between the indicated groups (exact permutation test, *p* = 0.10, the minimum achievable value for *n* = 3). For all time points marked with a dagger, effect sizes were large (Cohen’s *d* > 0.8), indicating biologically meaningful effects.

Figure 3 shows the average emergence rate of the female–male isolation test groups. Under yellow light treatment, the emergence rate of females was consistently lower than that of the control group throughout most of the observation period. Exact permutation tests revealed complete separation between treatment and control groups in females on days 2–4, 6, 8, 10, and 13 (Figure 3), indicating a clear distinction despite the small sample size. For males, the emergence rate was also generally lower than the control group, with complete separation observed on days 10 and 13.

Cohen’s d effect sizes for all time points showing complete separation were greater than 0.8, indicating large and biologically meaningful effects. In contrast, the original *t*-tests showed significant differences only on days 2–4 for females and day 13 for males (*p* < 0.05), which is consistent with the more conservative nature of exact permutation tests.

Comparisons between females and males revealed that females were more sensitive to yellow light treatment. Under control conditions, complete separation between females and males was observed on days 6–8 and 10, while under treatment conditions, complete separation occurred on days 5–8. Effect sizes for these comparisons were also large (*d* > 0.8). Overall, these results demonstrate that female *A. corpulenta* are more sensitive to yellow light than males, with stronger and more frequent treatment effects across the observation period.

**Figure 4:**
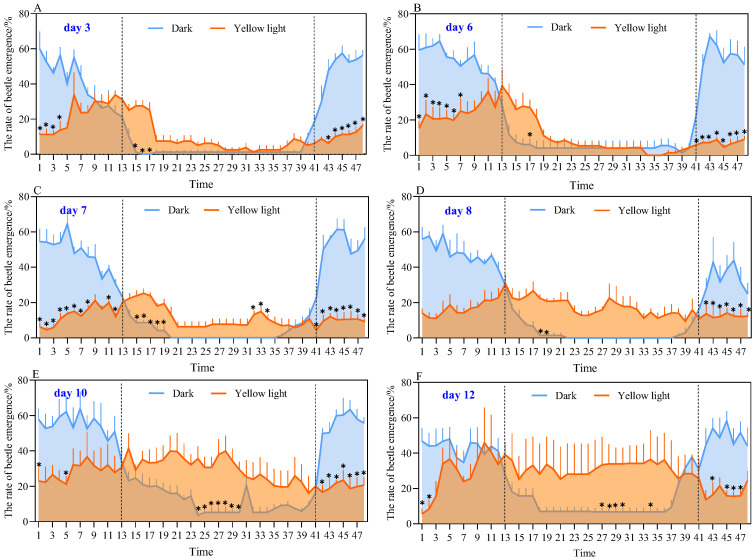
Emergence rate and rhythm of *Anomala corpulenta* (female-male mixed test group). Panels (**A**–**F**) show the emergence rates for the yellow light-treated group (orange) and the control group (blue). Data are shown as mean ± SE (*n* = 4). The shaded area under the curve is for visual guidance. Dashed vertical lines separate different phases of emergence rhythm. Values 1, 2 … 47, 48 on the horizontal axis represent time points from 00:00, 00:30 … to 23:00, 23:30, respectively. Asterisks (*) indicate significant differences between treatment and control groups as determined by exact permutation test (*p* < 0.05).

The emergence rate and rhythm of the mixed female-male test group are shown in Figure 4. During the 2nd–7th days of the experiment, the emergence pattern was similar to that of the female-male isolation test group. Compared with the control group, which exhibited an emergence peak between 20:00–24:00, the emergence peak of the experimental group was mainly concentrated between 00:00–06:00. Both exact permutation tests and *t*-tests revealed significant differences between the treatment and control groups at multiple time points during this period (*p* < 0.05) (Figure 4A–C), and effect sizes were also large (Cohen’s *d* > 0.8) for these significant time points, indicating large and biologically meaningful effects.

From the 8th day onward, the emergence peak of the experimental group gradually shifted backward, became more dispersed, and exhibited multiple peaks, with obvious emergence during the light period. The emergence rate was higher than that of the control group, with significant differences detected by both exact permutation tests and *t*-tests at multiple time points (*p* < 0.05) (Figure 4D–F). Effect sizes for these significant time points were also large (*d* > 0.8).

Overall, these results demonstrate that continuous exposure to yellow light significantly alters the emergence rhythm of *A. corpulenta*, while also impacting emergence rate. The consistency among *t*-tests, exact permutation tests, and effect size analysis confirms that the observed differences are not only statistically significant but also biologically meaningful.

**Figure 5:**
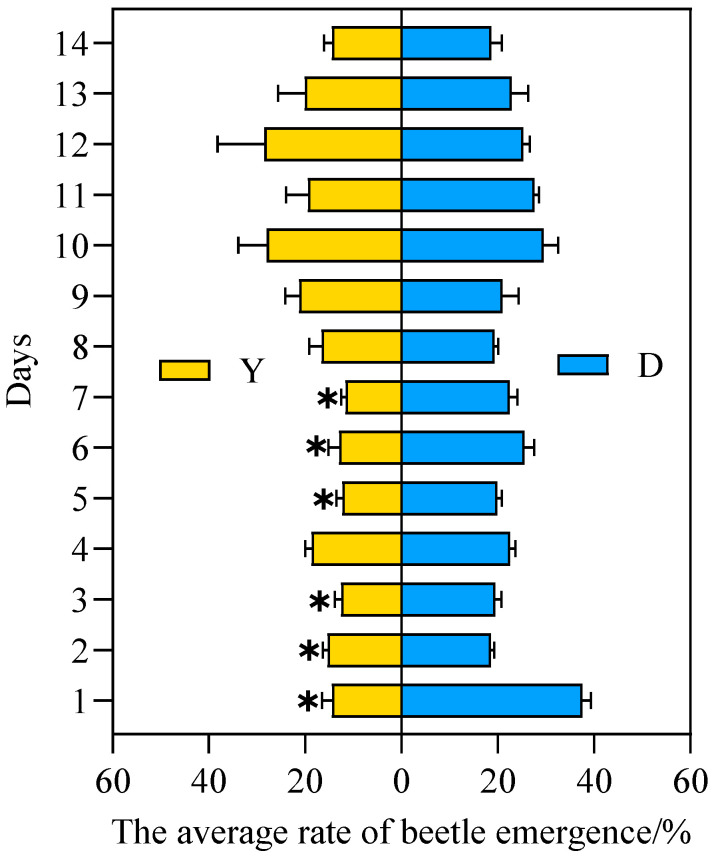
Mean emergence rate of male and female *Anomala corpulenta* (female-male mixed test group). Data are shown as mean ± SE. Asterisks (*) indicate significant differences between treatment and control groups as determined by exact permutation test (*p* < 0.05).

Figure 5 shows the average emergence rate of the mixed female-male test group, where the average emergence rate of the *A. corpulenta* experimental group was lower than that of the control group in all testing days, with both exact permutation tests and *t*-tests revealing significant differences on days 1–3 and 5–7 days (*p* < 0.05) (Figure 5). Effect sizes were also large (*d* > 0.8) for these significant time points, indicating large and biologically meaningful effects. Thus, continuous exposure to yellow light has a significant and biologically meaningful effect on the average emergence rate of *A. corpulenta.*

### 3.2. Effects of Continuous Exposure to Yellow Light on the Feeding and Mating of A. corpulenta

**Figure 6:**
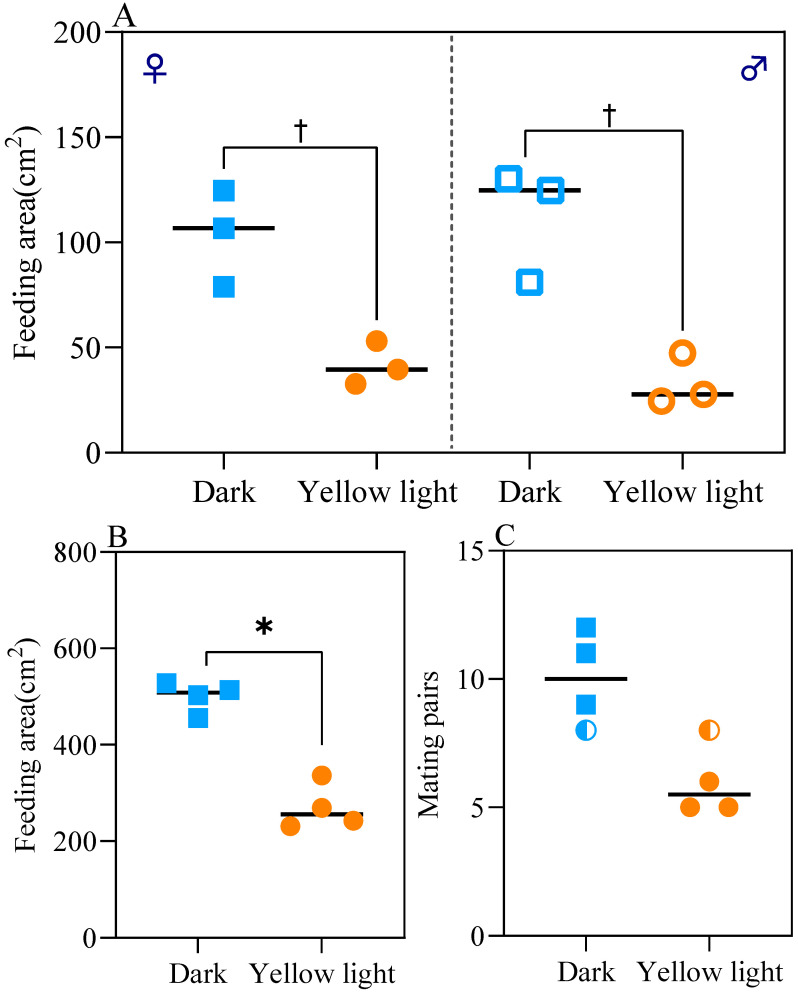
Feeding area and mating pair number of *Anomala corpulenta*. Data are shown as individual data points. Blue symbols represent the control group; orange symbols represent the yellow light-treated group. (**A**) Feeding area of isolated female and male populations; (**B**) Feeding area of mixed-sex population; (**C**) Number of mating pairs. Asterisks (*) indicate significant differences between treatment and control groups as determined by exact permutation test (*p* < 0.05). Daggers (†) indicate complete separation between treatment and control groups (exact permutation test, *p* = 0.10, the minimum achievable value for *n =* 3), with large effect sizes (Cohen’s *d* > 0.8), indicating biologically meaningful effects.

Statistical analyses were performed on the total feeding amount and number of mating pairs of *A. corpulenta* in the treatment and control groups, with the results presented in Figure 6.

In the female–male isolated test groups (*n* = 3, Figure 6A), the total feeding area of *A. corpulenta* in the experimental groups was markedly lower than that of the control groups. Independent samples *t*-tests revealed significant differences between treatment and control groups for both females and males (*p* < 0.05). Exact permutation tests further confirmed complete separation between treatment and control groups for both sexes (*p* = 0.10, the minimum achievable value for *n* = 3), and Cohen’s d effect sizes for these comparisons were large (*d* > 0.8). In the female-only test group, the total feeding area decreased from 103.37 cm^2^ (control) to 41.85 cm^2^, a reduction of 59.51%; in the male-only test group, it decreased from 112.02 cm^2^ (control) to 33.25 cm^2^, a reduction of 70.32% (Figure 6A). No significant difference was detected between males and females by any of the three statistical methods (*t*-test, exact permutation test, or Cohen’s d), and the effect size was small (*p* > 0.05; *d* < 0.2) (Figure 6A).

In the female–male mixed test group (*n* = 4, Figure 6B), the total feeding amount decreased from 500.08 cm^2^ (control) to 269.75 cm^2^, a reduction of 46.06%. Both exact permutation tests and *t*-tests revealed significant differences (*p* < 0.05), and effect sizes were also large (*d* > 0.8).

The number of mating pairs of *A. corpulenta* in the experimental group was lower than that of the control group, decreasing from 10 pairs in the control group to 6 pairs in the experimental group (a reduction of 40%). Independent samples *t*-tests revealed a significant difference between the two groups (*t* = −3.464, *df* = 6, *p* = 0.013). Specifically, the number of mating pairs decreased from 10 (control group) to 6, a significant decrease of 40%. However, due to the small sample size (*n =* 4), exact permutation tests were also conducted and yielded a non-significant result (exact *p* = 0.057), which is close to the conventional significance threshold. Cohen’s d effect size for this comparison was large (*d* > 0.8), indicating that the observed difference is biologically meaningful despite not reaching statistical significance under the more stringent permutation test (Figure 6C).

Collectively, these results demonstrate that continuous yellow light exposure significantly reduces the total feeding amount and number of mating pairs of *A. corpulenta*, with the magnitude of the effect being biologically meaningful.

### 3.3. Effect of Continuous Exposure to Yellow Light on the Longevity of A. corpulenta

**Figure 7:**
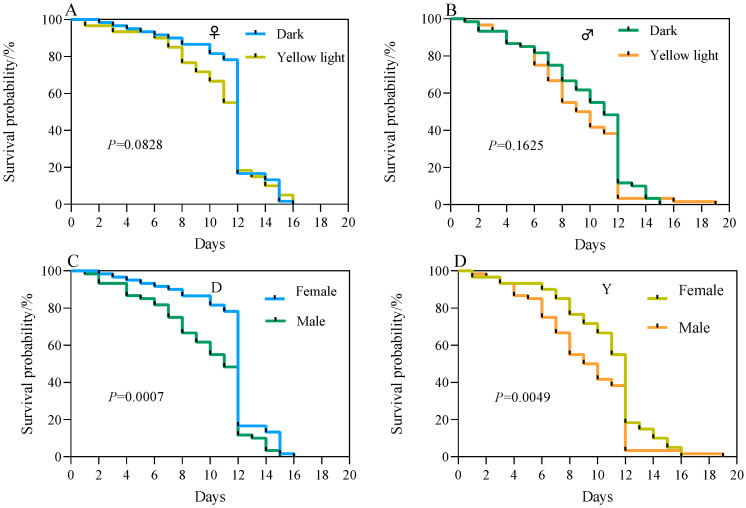
Survival curve of *Anomala corpulenta* (female-male isolation test group). (**A**) Comparison of survival probability between treatment and control groups in females; (**B**) Comparison of survival probability between treatment and control groups in males; (**C**) Comparison of survival probability between females and males in the control group; (**D**) Comparison of survival probability between females and males in the treatment group. Survival curves were compared using the Log-rank test.

**Figure 8:**
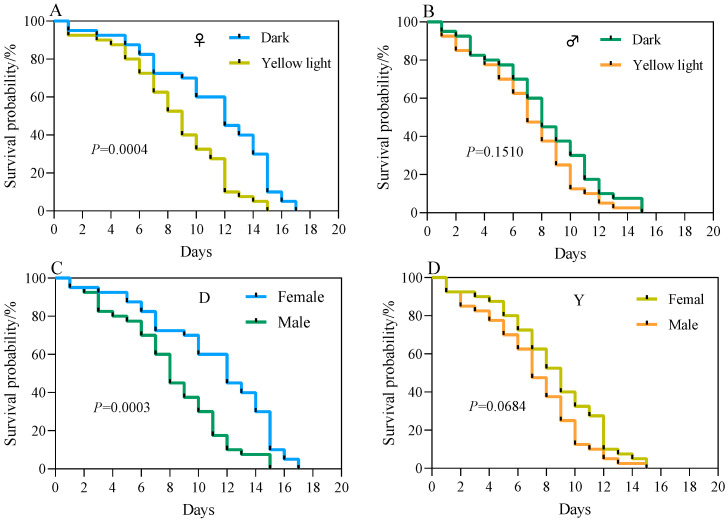
Survival curve of *Anomala corpulenta* (female-male mixed test group). (**A**) Comparison of survival probability between treatment and control groups in females; (**B**) Comparison of survival probability between treatment and control groups in males; (**C**) Comparison of survival probability between females and males in the control group; (**D**) Comparison of survival probability between females and males in the treatment group. Survival curves were compared using the Log-rank test.

Survival tests revealed no significant difference in the survival probabilities of male and female adult *A. corpulenta* between the experimental and the control group in the female–male isolated trials (*p* > 0.05) (Figure 7A,B). In the female–male mixed test group, however, the survival probability of female adult *A. corpulenta* was significantly lower in the experimental group than in the control group (*p* < 0.05) (Figure 8A). No significant difference was observed in the survival probability of male adults between these two groups in the same mixed populations (*p* > 0.05) (Figure 8B).

In the female-male isolated trials, the survival probability of males was significantly lower than that of females in both the control and experimental groups (*p* < 0.05) (Figure 7C,D). In the female-male mixed trials, however, a significantly lower survival probability was observed in males relative to females in the control group (*p* < 0.05), whereas no significant difference was detected in survival probability between males and females in the experimental group (*p* > 0.05) (Figure 8C,D).

## 4. Discussion

Light is one of the most important environmental factors influencing insect behavior. An increasing number of studies have demonstrated that exposure to artificial light at night can disrupt the normal behaviors of nocturnal insects, including flight, orientation, dispersal, migration, communication, predation, mate recognition, oviposition, eclosion, molting, and various rhythmic activities [8,27,28,29,30]. Based on this characteristic, employing artificial light with specific wavelengths, intensities, and irradiation patterns to interfere with or regulate key behavioral processes of pests has emerged as a promising technical approach in the field of physical pest control. Earlier studies in Japan demonstrated that yellow light (approximately 580 nm) could inhibit the activity of fruit-piercing moths and other noctuid pests, likely by maintaining their compound eyes in a light-adapted state, thereby reducing feeding and reproductive behaviors [31,32,33]. For instance, Yase et al. reported that overnight illumination with yellow fluorescent lamps reduced feeding damage by *Helicoverpa armigera*, *S. exigua*, and *S. litura* on ornamental crops. Similarly, Yoon et al. observed that yellow LED lighting disrupted the flight activity of *H. armigera* [33,34]. Continuous exposure of moths to yellow and green light at night maintains their compound eyes in a ‘light adaptation state’, thereby interfering with their natural rhythms and adversely affecting foraging, mating, and oviposition. This disruption may reduce the lifespan and reproductive capacity of adults, potentially contributing to population control. Yellow lamps utilize this principle for the management of moth pests [15,16,32,33,34,35,36,37,38]. These studies provide important references and insights for the use of specific spectra to manage other pest populations. Previous research has shown that under the stress of yellow and green light exposure, the emergence rate of *A. corpulenta* and *Holotrichia* (Motschulsky, 1854) decreased notably, and their emergence rhythms, feeding, and mating activities were significantly affected [21,39]. While those results preliminarily clarified the effects of short-term exposure to yellow and green light on the behavior of *A. corpulenta*, evidence supporting the use of yellow light for controlling this pest remained insufficient. Given that short-term effects may not fully reflect the actual conditions of long-term field applications, and pests might develop adaptive behaviors or physiological compensations, it is essential to further elucidate the impacts of continuous exposure to yellow light on key behavioral activities of *A. corpulenta*, including emergence rhythm, emergence rate, feeding, and mating. Understanding this is crucial for assessing its potential long-term effectiveness and ecological safety as a sustainable control strategy.

The current study demonstrated that continuous exposure to yellow light significantly altered the emergence rhythm of *A. corpulenta*, irrespective of whether the insects were subjected to female-male isolated or mixed trials. With prolonged exposure duration, the emergence peak was delayed and became more dispersed, resulting in multiple peaks, with a notably distinct emergence peak occurring during the light period. This disruption and desynchronization of rhythm may suggest that continuous light exposure interferes with the coupling between the insect’s internal circadian clock and external light-dark environmental signals, leading to disturbances in its temporal organization. Additionally, exposure to yellow light significantly affected the emergence rate; during specific time periods, the emergence rate in the experimental group was notably lower than that in the control group. Moreover, the average emergence rate during the initial testing phase decreased compared to the control group. This reduction in emergence rate could be related to the insect’s avoidance behavior toward potentially risky environments, or it might result from altered energy allocation—where increased energy expenditure to cope with light exposure could lead to reduced energy available for activities such as emergence.

Changes in emergence rhythm and rate during the first five test days aligned closely with diurnal variations reported in previous studies [21]. This study confirms the validity of short-term experimental results during the initial phase of continuous treatment and supports the coherence of the experiment. Furthermore, prolonged exposure to yellow light significantly reduced both the total food intake and mating pairs of *A. corpulenta*, directly impacting its energy intake and reproductive success—key factors in controlling population growth. The observed decrease in food consumption could be attributed to reduced foraging activity due to light exposure, an impaired ability to locate host plants, or indirect effects on digestive physiology. The suppression of mating behavior might stem from light interference with normal pheromone communication, decreased opportunities for mate encounters, or disruptions in endocrine balance related to reproduction. Additionally, yellow light also influenced the lifespan of the beetles. Importantly, the conclusions of this study align with findings from related research on Lepidoptera pests [15,20,33,36]. This suggests that nocturnal insects from different orders (such as Lepidoptera and Coleoptera) may exhibit common physiological and behavioral response patterns to specific wavelengths of artificial light, despite variations in their visual systems and ecological niches.

The phenomenon of sexual dimorphism in phototaxis among adult males and females is widespread in nature. While different insect species exhibit distinct phototactic patterns, adult males and females of the same species may also display differential phototactic responses under varying conditions [40]. This sexual dimorphism could be closely related to distinct life history strategies, energy requirements, and reproductive roles; for instance, females need to locate suitable oviposition sites while males actively seek mates. Current findings indicate that female *A. corpulenta* exhibit more pronounced changes in emergence rate and circadian rhythm under yellow light exposure than males. This suggests that females may be more sensitive to variations in their light environment or that their emergence behavior is more strictly regulated by endogenous rhythms, rendering them more susceptible to disturbance. In contrast, male lifespan was more significantly affected by yellow light exposure. This could be attributed to males adopting more active yet energetically costly behavioral strategies when coping with stress, or to their relatively lower physiological tolerance. These results indicate that continuous exposure to yellow light differentially affects the biological behaviors of male and female *A. corpulenta* adults. Therefore, when evaluating the population-level effects of light-based control technologies and formulating control strategies, it may be crucial to consider sex-specific responses, as their impact on reproductive potential is mediated through distinct behavioral responses of both sexes.

To date, extensive research and practical application have focused on the control efficacy of yellow light on nocturnal moth pests [8,17]. Yellow light traps have been advocated as part of physical control measures in agricultural practices. However, relatively few studies have examined the efficacy of yellow light in controlling nocturnal Coleoptera insects. As the largest order within the class Insecta, Coleoptera includes numerous significant agricultural and forestry pests, as well as beneficial insects, making it both theoretically and practically important to elucidate the effects of light on their behavior. Our preliminary research explored the effects of yellow light on the behavioral activities of *A. corpulenta* and *H. parallela*. The present study further investigated the impact of prolonged exposure to yellow light on *A. corpulenta*, with results indicating that sustained yellow light irradiation significantly altered its emergence rhythm, reduced emergence rates, and markedly decreased total food consumption and mating frequency, thereby potentially impairing its reproductive capacity and suppressing population growth [17]. This cascade of effects forms a comprehensive chain linking individual behavioral disruptions to the suppression of population growth.

From a practical perspective, these findings offer insights for yellow light-based pest management. Unlike trapping devices, this approach functions by continuously interfering with adult beetle behavior. In field applications, yellow lights would be installed at approximately 2 m above ground with a density of 2 lamps per mu. Given that continuous exposure significantly suppressed feeding, mating, and emergence, deploying such lights during peak adult activity periods could reduce population density. Field studies have reported 55.54–72.11% control efficacy against moth pests using 589 ± 5 nm yellow lights [14], suggesting similar potential for *A. corpulenta*, consistent with earlier Japanese studies on noctuid pests [31,32,33,34]. Based on observed sex-specific responses, implementation during early adult emergence may be most effective. Integrating yellow light with other IPM components, such as pheromone lures, could enhance suppression while reducing chemical reliance [1]. Regarding non-target effects, recent field evaluations suggest that 589 ± 5 nm yellow lights have no significant adverse effects on non-target insects or natural enemies [14], likely due to the specific wavelength used. Nevertheless, further long-term studies across diverse agroecosystems are warranted to fully assess ecological safety.

While the results of this study are encouraging, certain limitations should be acknowledged. The sample size in each experimental group was relatively small (*n =* 3 or 4), which may affect the generalizability of the findings. Therefore, further studies with larger sample sizes are needed to confirm these observations and to better understand the variability of responses under different environmental conditions.

The findings theoretically enrich the field of insect visual ecology, particularly by expanding our understanding of phototactic behaviors in non-lepidopteran insects. Additionally, this research provides new case studies that enhance our comprehension of how artificial light impacts the ecology of subterranean pests. Furthermore, it offers preliminary guidance for managing *A. corpulenta* through the use of yellow light, thereby supporting the feasibility of employing specific-wavelength light sources as an environmentally friendly technological option within IPM systems for scarabaeid pests. However, the mechanisms underlying the effects of continuous yellow light exposure on *A. corpulenta* remain unclear, and could involve interference with circadian rhythms, or a possible repellent effect. More complex explanations are also plausible. For instance, light stress might influence insects’ physiological states through non-visual pathways, such as affecting hormone secretion (e.g., melatonin), or indirectly impact their behavior by altering energy metabolism and oxidative stress levels. Furthermore, future research should integrate multidisciplinary approaches from neurobiology, molecular ecology, and field ecology. This could involve an in-depth exploration across multiple dimensions, including photoreceptor types, circadian clock gene expression, energy metabolic profiles, and population dynamic models, to comprehensively elucidate the underlying mechanisms. Additionally, optimizing light parameters—such as intensity, duration, and wavelength combinations—in conjunction with field validation studies across different crops and geographic regions, will be important for achieving efficient and precise ecological light regulation while ensuring the long-term sustainability and ecological compatibility of this approach.

## 5. Conclusions

This study demonstrates that continuous nocturnal exposure to yellow light (565–585 nm, 30–40 lx) profoundly disrupts key behavioral processes in *A. corpulenta*, a major agricultural pest. Prolonged irradiation significantly altered emergence rhythms, characterized by delayed, dispersed, and multiple emergence peaks, including emergence during the photophase. Concurrently, feeding activity and mating frequency were markedly suppressed, with reductions of up to 70% in food consumption and 40% in mating pairs, directly impacting energy intake and reproductive potential. Notably, females exhibited greater phototactic sensitivity than males, with more pronounced and frequent behavioral disruptions. Importantly, the congruence among *t*-tests, exact permutation tests, and large effect sizes (Cohen’s *d* > 0.8) confirms that these effects are not only statistically robust but also biologically meaningful. These findings provide a mechanistic basis for developing wavelength-specific lighting as an environmentally sustainable strategy for pest management, while advancing our understanding of insect visual ecology and the differential behavioral responses between sexes under artificial light stress.

## Data Availability

The original contributions presented in this study are included in the article. Further inquiries can be directed to the corresponding authors.
